# South African Institute of Drug-Free Sport Position Statement on CBD (Cannabidiol) and THC (Tetrahydrocannabinol)

**DOI:** 10.17159/2078-516X/2023/v35i1a16097

**Published:** 2023-10-02

**Authors:** L Pillay, C Thompson, C Tabane, J Kirby, S Hendricks, J Swart, DC Janse van Rensburg, P Zondi, A Rotunno, D Bayever

**Affiliations:** 1 South African Institute for Drug-free Sport (SAIDS), Sport Science Institute of South Africa, 4th Floor, Newlands, Cape Town, South Africa; 2 South African Sports Medicine Association (SASMA), 668 Corelli Street, Les Marais Pretoria, South Africa; 3 International Federation of Sports Medicine (FIMS), Maison du Sport International, Av. de Rhodanie 54, Lausanne, Switzerland; 4 Institute of Sport and Exercise Medicine, Department of Exercise, Sport and Lifestyle Medicine, Faculty of Medicine of Health Sciences, Stellenbosch University, South Africa; 5 HPALS Research Center, Division of Physiological Sciences, Department of Human Biology, University of Cape Town, Cape Town, South Africa; 6 Section Sports Medicine, Faculty of Health Sciences, University of Pretoria, Pretoria, South Africa; 7 Faculty of Health, University of Witwatersrand, Johannesburg, South Africa

**Keywords:** CBD, THC, athletes, cannabis, doping

## Abstract

Cannabidiol (CBD) and Tetrahydrocannabinol (THC) have become easily available to athletes over the years. Using these substances may inadvertently expose an athlete to the possibility of an adverse analytical finding (a ”positive” test) and a sanction. Athletes need to understand the risk of an anti-doping rule violation or adverse analytical finding should these products be used, especially if no therapeutic use exemption exists. This position statement attempts to clarify the use of CBD and THC and their associated risks with Anti-Doping Rule Violations (ADRV) in the athletic population. The South African Sports Medicine Association supports this position statement.

In recent years, there has been an increasing debate and legislative review related to Cannabidiol (CBD) and Tetrahydrocannabinol (THC). ^[1;2;3]^ CBD and THC are the active ingredients in the cannabis plant, commonly known as marijuana. ^[4]^ Due to various health concerns and its neurological effects, especially the behavioural component (e.g. temporary neurocognitive dysfunction and delayed physical responsiveness), it is prohibited in most sporting codes and considered an illegitimate recreational substance in many countries. ^[3;4;5]^ The recent move toward legalising CBD and THC in some countries or regional states has gained significant traction, with proponents advocating for regulatory changes that will enable (1) personal use, (2) medicinal use and (3) cultivation. In countries where the substance is decriminalised, these products are readily available at specialist distributors, chain stores, pharmacies and online resellers. This availability to the general public may expose the athletic population to its potential use, which may result in adverse analytical findings in doping control tests (a “positive test”). These products are marketed as treatment options for various medical conditions including, but not limited to, stress, anxiety, chronic pain syndromes, skin conditions, sleep disorders, cancer chemotherapy, and nausea. ^[5;6]^ In sport, the use of cannabis is prohibited in-competition and classified as a Substance (S8) on the World Anti-Doping Code (WADC) International Standard Prohibited List for 2023. ^[7]^ As regulations related to legality and use are reviewed country by country, it is important to establish a view from a National Anti-Doping Organisation (NADO). This will assist athletes in making informed decisions about the personal use of cannabis while helping them to understand the potential risks related to sport participation.

## What is CBD?

CBD is one of the compounds found in the cannabis plant. ^[4]^ It is a popular natural remedy for several medical conditions (e.g. anxiety and insomnia) and an essential component of medical cannabis. ^[1;4]^ It does not possess the euphoric and addictive side effects compared to THC-containing products but may produce other side effects, e.g. xerostomia (dry mouth), diarrhoea, fatigue and somnolence. ^[8]^

## What is THC?

THC is the psychoactive compound ^[4]^ found in the cannabis plant, which is responsible for the euphoric feeling (commonly referred to as a “high”) that results from ingestion or inhalation of cannabis. The scientific literature still cannot explain the effect of euphoria thoroughly, as it is a complex relationship between neurotransmitters and receptors in the brain. This ingredient may lead to dependence and abuse of cannabis. ^[9;10]^ It is also considered a gateway drug that may lead to the use and abuse of other substances. ^[11]^

## What formulations of CBD and THC are available?

There are various formulations of CBD and THC products on the market. Each has its own delivery method and potential side effects. ^[12]^ Some examples include:

1. *Capsules/tablets:* tablets or capsules containing measured doses of CBD or THC. As the digestive tract absorbs them, the onset of their effect may be delayed, and these effects may be more prolonged than other formulations.
2. *Edible forms:* there are numerous types of edible CBD or THC-infused products, including baked goods, chocolates, sweets, etc. Again, due to digestion, the onset of their effect may be delayed, and these effects may be more prolonged. There is a considerable inter-individual variation in digestion and metabolism rates, and it is difficult to quantify the severity and duration of the pharmacological effect. ^[4]^
3. *Inhaling/E-cigarettes:* inhalational forms of CBD or THC. These are available in cartridges (disposable or rechargeable) or rolled in paper or smoked from pipes, and due to the quick absorption by the lungs, the onset of action is generally rapid. There may also be other additives with the potential of addictive or harmful properties.
4. *Topical:* creams, ointments, balms or lotions with CBD or THC can be applied topically to the skin for potential pain relief.
5. *Transdermal patches:* CBD or THC-infused patches are applied to the skin and deliver a controlled dose over a period of time.
6. *Concentrates/tinctures:* extracts of CBD or THC that are used in addition to foods, liquids, vape cartridges, or sublingually (under the tongue).

Given the differing inter-individual side effects and potencies, the user must be familiar with the various formulations and their relative indications for use. It is prudent for the athlete to consult a knowledgeable medical professional before using any of these products.

## If it is illegal, why is it available at pharmacies?

Historically, cannabis was illegal in many countries, including South Africa (SA). In September 2018, the South African Constitutional Court found the criminalisation of cannabis for home use and cultivation unconstitutional. ^[13]^ Subsequently, SA formulated specific legislation to decriminalise cannabis use and cultivation. The legislation is complex and currently being approved in stages by the South African parliament. It addresses aspects related to manufacturing, quality control, and distribution. It must align with relevant regulatory bodies such as the South African Health Product Regulatory Association (SAHPRA) and key policies such as the Good Manufacturing Practice (GMP). ^[2;13]^ Since becoming partially legislated in 2018, many pharmacies in South Africa have sold CBD, but only at permissible, regulated concentrations as an over-the-counter product without needing a prescription. ^[1;2]^ The South African Health Product Regulatory Authority has approved a few pharmacies to resell THC products under prescription from a healthcare provider. Some countries are still to legalise cannabis; therefore, any product consumer must be mindful of this when travelling and knowledge of the relevant local CBD or THC regulations are important.

## The World Anti-Doping Agency’s (WADA) views on CBD and THC

Listed in the S8 category, cannabis is considered a prohibited substance by the World Anti-Doping Agency (WADA). It is expressly prohibited in competition. However, the World Anti-Doping Agency has acknowledged the increasing availability of CBD or THC in various nations and tasked their List Expert Advisory Group (LiEAG) and various scientific units to explore the health and performance consequences in athletes. As a result of some of this research ^[14;15]^, the World Anti-Doping Agency, in 2021, classified cannabis as a Substance of Abuse. This research mostly found the effects on mental health and general health consequences of the use of cannabis. A substance of abuse has an addictive nature and regular use may affect an athlete’s general health. In 2023, the threshold limit for THC in urine changed to 150ng/ml to trigger an Adverse Analytical Finding (AAF) and 180ng/ml to decide on sanction. This means that should the urine analysis show a THC concentration of 150ng/ml, this will be reported as a “positive” test for THC. If the concentration is over 180ng/ml, this will determine the method, manner and period of sanction. Cannabidiol (CBD) is an exception and is not prohibited as it has no psychoactive properties. The athlete must know how or why a particular substance is prohibited in sports. According to WADA, the substance must fulfil any two or more of the following criteria to be considered banned:

1. The substance has the potential to, or enhance sports performance.
2. The substance represents a potential or actual health risk to the individual.
3. The substance violates the spirit of sport.

After reviewing the evidence, WADA took the following stance on THC in September 2022:

“After a thorough assessment and discussion under WADA Code Article 4.3, the List Expert Advisory Group concluded that:

- There is compelling medical evidence that the Use of THC is a risk for health, mainly neurological, which has a significant impact on the health of young individuals, a cohort which is overrepresented in athletes.
- The current body of objective evidence does not support THC enhancement of physiological performance, while the potential for performance enhancement through neuropsychological effects still cannot be excluded.
- In consideration of the values encompassed by the Spirit of Sport as outlined by the Ethics Expert Advisory Group, and noting that respect for self and other participants includes fellow competitors’ safety, the Use of THC in In-competition violates the Spirit of Sport.

Based on these three criteria defined by the Code, on the scientific evidence available, THC meets the criteria to be included on the List.”

## How strong is the evidence for the use of CBD and THC?

Several studies relate to using CBD, THC or a combination thereof. However, these studies have a low level of evidence, e.g. no randomised controlled trials, small sample sizes or case studies. ^[4;16;17;18;19]^ One of the recognised techniques to evaluate the efficacy of an intervention is a study process defined as a “Systematic review”. This involves a review of the available scientific literature pertaining to the topic. The literature is then critically evaluated to assess the methods used and the quality of the evidence presented. The evidence is then graded accordingly and a summary of the strength of the evidence is presented. Recent systematic reviews have insufficient scientific evidence to substantiate using these preparations as a first-line agent in managing medical conditions. ^[4;16;17;18;19]^ This is evident in the realms of utilising cannabis for its use in determining the ratio of CBD to THC in preparations ^[4]^, in psychiatric disorders ^[16]^, chronic pain ^[17]^ sleep disorders ^[18]^ and the oncology patient as discussed below. ^[19]^

## What conditions are commonly treated using CBD and THC?

Some research suggests that CBD and THC may be effective is the following conditions:

1. Anxiety ^[16]^
2. Pain management ^[17]^
3. Sleep disorders ^[18]^
4. Oncology chemotherapy side-effect management ^[19]^

This list is not exhaustive and only represents the most common clinical conditions known to be treated using CBD and THC.

## Athlete considerations when using CBD and THC-containing products

Cannabis currently appears on the World Anti-Doing Code International Standard Prohibited List for 2023. ^[20]^ The chemical compound THC is the main active component screened for and may produce an adverse analytical finding if detected. The chemical compound CBD is not a prohibited substance. However, the South African Health Products Regulatory Authority allows a small percentage of THC in the manufacture of CBD-containing products. Therefore, there is a possibility of unintentional doping. At present, there are no pharmacokinetic studies that have definitively determined whether the long-term use of CBD can result in a positive drug test.

The detection of cannabis use in urine samples varies depending on the frequency of use. Generally, single use is detectable for 2 days, 3 x a week for 2 weeks, daily use for 2 to 4 weeks and heavy use for 4 to 6 weeks (up to 12 weeks) ^[21]^. A “typical” cannabis cigarette once-off consumption may increase levels to 100ng/ml in 15 minutes but dissipate to 2ng/ml within 4 hours ^[22]^. Chronic consumption may increase these levels even further. It’s crucial for cannabis users to be aware of their frequency of use. The concentration of cannabis in products can vary, depending on how it’s consumed (smoked, vaporized, or consumed), individual physiological states, and frequency of use. Due to these factors, it’s difficult to accurately determine the concentration of cannabis that will result in a urine concentration level of 150ng/ml or higher. Secondary exposure may increase your cannabis exposure (25μg/m^3^–53μg/m^3^ for a puff frequency of one a day), especially if this is on a chronic basis. ^[23]^ This may lead to levels above the threshold of 150ng/ml.

## Guidance for athletes

Many athletes will be familiar with the term “strict liability”. This means that it is the athlete’s sole responsibility to ensure that they are not using prohibited substances, whether self-administered, prescribed by a healthcare practitioner, or issued over the counter. The athlete must check all medication, supplements or products used against the World Anti-Doping Code International Standard Prohibited List to ensure they are safe to use in sports. Should there be any adverse analytical finding, the athlete will be held accountable regardless of whether a healthcare professional prescribed the medication, supplement or product. With this said a list of medical conditions requiring medication that appears on the prohibited list. If a healthcare practitioner has diagnosed any of these conditions in an athlete, they must provide clinical evidence and test results to confirm the diagnosis and rationale for use. The athlete would use this information to apply for Therapeutic Use Exemption (TUE) or “permission” from their National Anti-Doping Organisation before commencing treatment. ^[24;25]^ Only under particular circumstances and rules will retrospective therapeutic use exemptions be accepted. It is imperative to note that all therapeutic use exemptions applications undergo comprehensive assessment and scrutiny by an expert panel, who will only approve applications that meet strict clinical criteria (www.drugfreesport.org.za/tue/). Physician guidelines and checklists are available to compare evidence against the World Anti-Doping Agency requirements. ^[26]^

## Considerations if you suffer from any medical condition for which CBD and THC are used or prescribed

In all the conditions listed, alternative first-line treatment options that do not require the use of prohibited substances are available. Athletes and their healthcare providers are encouraged to explore these options first and compare these substances against the Prohibited List and the World Anti-Doping Agency’s medical practitioner guidance in treating particular conditions. ^[26]^

*Anxiety and related disorders* ^[16]^*:* A patient must meet certain diagnostic criteria according to the Diagnostic and Statistical Manual (DSM V) ^[27]^ for diagnosing a psychiatric disorder. Once a clinician confirms the diagnosis, psychotherapy and behavioural therapy are the cornerstones of management. Medication should only be prescribed by a healthcare professional under strict medical guidance.

*Pain* ^[17]^*:* Pain is a common symptom experienced by athletes for various reasons. Pain management is a complex clinical scenario requiring a holistic multi-disciplinary team approach (doctors, psychologists, biokineticists, physiotherapists). Physical therapy, electrical stimulation and ultrasound may be utilised, together with medication, under medical guidance depending on the aetiology of pain.

*Sleep disorders* ^[18]^*:* it is necessary first to establish the nature of the sleep disorder and conduct a thorough history, physical examination, and behavioural and risk assessment. The cornerstone of management involves treating the root cause and may require psychotherapy or behavioural therapy. Medication is only sometimes necessary and should only be prescribed by a medical doctor.

*Chemotherapy side effects* ^[19]^*:* Athletes may also become oncology patients. Some chemotherapeutic medications may have severe side effects (like nausea, body pains etc.), which must be managed with prescription medication accordingly. An athlete’s health must always be prioritised over any performance objectives. Frank and honest discussion with the treating oncologist is required about the best management method for pain, nausea, fatigue and insomnia. Once an athlete has recovered, is in regression and is ready to train and compete, the athlete must ensure any medication still being used is not prohibited in or out of competition. This can be done by checking the medication by trade name on the South African Institute of Drug-Free Sport website medication check online function (https://drugfreesport.org.za/online-medication-check/), the World Anti-Doping Agency Prohibited List (via www.wada-ama.org) or GlobalDRO (www.globaldro.com/home).

In all cases where cannabinoid products are intended for “medical purposes” by an athlete, a definitive diagnosis should be forthcoming from the treating medical practitioner. In addition, appropriate, evidence-based, medically-guided treatment protocols should be implemented before opting to use methods and substances where the evidence is still not yet robust enough to prove efficacy and safety.

## Conclusion

Athletes must always consider medication, supplements or products and their position on the World Anti-Doping Code Prohibited List before use. Using a prohibited substance with subsequent doping control resulting in an adverse analytical finding without a valid Therapeutic Use Exemption may invite legal proceedings and sport participation sanctions, leading to undue stressors. Relating to cannabis, the treating and prescribing physician must ensure they have enough medical evidence and have exhausted all other management aspect before its use to avoid a legal sanction proceeding after a possible adverse analytical finding. Physicians need to ensure they have clinical information that can be shared, displaying the use of evidence-based approaches that have failed and rational reasoning as to why cannabis should be considered. It is important to note that a Therapeutic Use exemption application does not grant automatic approval and is scrutinised by the Therapeutic Use Exemption Commission regarding its validity.
